# Evaluating the Digital Health Experience for Patients in Primary Care: Mixed Methods Study

**DOI:** 10.2196/50410

**Published:** 2024-04-11

**Authors:** Melinda Ada Choy, Kathleen O'Brien, Katelyn Barnes, Elizabeth Ann Sturgiss, Elizabeth Rieger, Kirsty Douglas

**Affiliations:** 1 School of Medicine and Psychology College of Health and Medicine The Australian National University Canberra Australia; 2 Academic Unit of General Practice Office of Professional Leadership and Education ACT Health Directorate Canberra Australia; 3 School of Primary and Allied Health Care Monash University Melbourne Australia

**Keywords:** digital health, eHealth, primary care, general practice, digital divide, health inequities, health inequality, disparities, digital cost, financial cost, health technology, mixed methods, barriers, barrier

## Abstract

**Background:**

The digital health divide for socioeconomic disadvantage describes a pattern in which patients considered socioeconomically disadvantaged, who are already marginalized through reduced access to face-to-face health care, are additionally hindered through less access to patient-initiated digital health. A comprehensive understanding of how patients with socioeconomic disadvantage access and experience digital health is essential for improving the digital health divide. Primary care patients, especially those with chronic disease, have experience of the stages of initial help seeking and self-management of their health, which renders them a key demographic for research on patient-initiated digital health access.

**Objective:**

This study aims to provide comprehensive primary mixed methods data on the patient experience of barriers to digital health access, with a focus on the digital health divide.

**Methods:**

We applied an exploratory mixed methods design to ensure that our survey was primarily shaped by the experiences of our interviewees. First, we qualitatively explored the experience of digital health for 19 patients with socioeconomic disadvantage and chronic disease and second, we quantitatively measured some of these findings by designing and administering a survey to 487 Australian general practice patients from 24 general practices.

**Results:**

In our qualitative first phase, the key barriers found to accessing digital health included (1) strong patient preference for human-based health services; (2) low trust in digital health services; (3) high financial costs of necessary tools, maintenance, and repairs; (4) poor publicly available internet access options; (5) reduced capacity to engage due to increased life pressures; and (6) low self-efficacy and confidence in using digital health. In our quantitative second phase, 31% (151/487) of the survey participants were found to have never used a form of digital health, while 10.7% (52/487) were low- to medium-frequency users and 48.5% (236/487) were high-frequency users. High-frequency users were more likely to be interested in digital health and had higher self-efficacy. Low-frequency users were more likely to report difficulty affording the financial costs needed for digital access.

**Conclusions:**

While general digital interest, financial cost, and digital health literacy and empowerment are clear factors in digital health access in a broad primary care population, the digital health divide is also facilitated in part by a stepped series of complex and cumulative barriers. Genuinely improving digital health access for 1 cohort or even 1 person requires a series of multiple different interventions tailored to specific sequential barriers. Within primary care, patient-centered care that continues to recognize the complex individual needs of, and barriers facing, each patient should be part of addressing the digital health divide.

## Introduction

### The Promise of eHealth

The rapid growth of digital health, sped up by the COVID-19 pandemic and associated lockdowns, brings the promise of improved health care efficiency, empowerment of consumers, and health care equity [1]. Digital health is the use of information and communication technology to improve health [2]. eHealth, which is a type of digital health, refers to the use of internet-based technology for health care and can be used by systems, providers, and patients [2]. At the time of this study (before COVID-19), examples of eHealth used by patients in Australia included searching for web-based health information, booking appointments on the web, participating in online peer-support health forums, using mobile phone health apps (mobile health), emailing health care providers, and patient portals for electronic health records.

Digital health is expected to improve chronic disease management and has already shown great potential in improving chronic disease health outcomes [3,4]. Just under half of the Australian population (47.3%) has at least 1 chronic disease [5]. Rates of chronic disease and complications from chronic disease are overrepresented among those with socioeconomic disadvantage [6]. Therefore, patients with chronic disease and socioeconomic disadvantage have a greater need for the potential benefits of digital health, such as an improvement in their health outcomes. However, there is a risk that those who could benefit most from digital health services are the least likely to receive them, exemplifying the inverse care law in the digital age by Hart [7].

### Our Current Understanding of the Digital Health Divide

While the rapid growth of digital health brings the promise of health care equity, it may also intensify existing inequities [8]. The digital health divide for socioeconomic disadvantage describes a pattern in which patients considered socioeconomically disadvantaged who are already marginalized through poor access to traditional health care are additionally hindered through poor access to digital health [9]. In Australia, only 67.4% of households in the lowest household income quintile have home internet access, compared to 86% of the general population and 96.9% of households in the highest household income quintile [10]. Survey-based studies have also shown that even with internet access, effective eHealth use is lower in populations considered disadvantaged, which speaks to broader barriers to digital health access [11].

The ongoing COVID-19 global pandemic has sped up digital health transitions with the rapid uptake of telephone and video consultations, e-prescription, and the ongoing rollout of e-mental health in Australia. These have supported the continuation of health care delivery while limiting physical contact and the pandemic spread; however, the early evidence shows that the digital health divide remains problematic. A rapid review identified challenges with reduced digital access and digital literacy among the older adults and racial and ethnic minority groups, which are both groups at greater health risk from COVID-19 infections [12]. An Australian population study showed that the rapid uptake of telehealth during peak pandemic was not uniform, with the older adults, very young, and those with limited English language proficiency having a lower uptake of general practitioner (GP) telehealth services [13].

To ensure that digital health improves health care outcome gaps, it is essential to better understand the nature and nuance of the digital health divide for socioeconomic disadvantage. The nature of the digital health divide for socioeconomic disadvantage has been explored primarily through quantitative survey data, some qualitative papers, a few mixed methods papers, and systematic reviews [11,14-16]. Identified barriers include a lack of physical hardware and adequate internet bandwidth, a reduced inclination to seek out digital health, and a low ability and confidence to use digital health effectively [16]. The few mixed methods studies that exist on the digital health divide generally triangulate quantitative and qualitative data on a specific disease type or population subgroup to draw a combined conclusion [17,18]. These studies have found digital health access to be associated with education, ethnicity, and gender as well as trust, complementary face-to-face services, and the desire for alternative sources of information [17,19].

### What This Work Adds

This project sought to extend previous research by using an exploratory mixed methods design to ensure that the first step and driver of our survey of a larger population was primarily shaped by the experiences of our interviewees within primary care. This differs from the triangulation method, which places the qualitative and quantitative data as equal first contributors to the findings and does not allow one type of data to determine the direction of the other [18]. We qualitatively explored the experience of digital health for patients with socioeconomic disadvantage and chronic disease and then quantitatively measured some of the qualitative findings via a survey of the Australian general practice patient population. Our key objective was to provide comprehensive primary mixed methods data, describing the experience and extent of barriers to accessing digital health and its benefits, with a focus on the digital health divide. We completed this research in a primary care context to investigate a diverse community-based population with conceivable reasons to seek digital help in managing their health. Findings from this mixed methods study were intended to provide health care providers and policy makers with a more detailed understanding of how specific barriers affect different aspects or steps of accessing digital health. Ultimately, understanding digital health access can influence the future design and implementation of digital health services by more effectively avoiding certain barriers or building in enablers to achieve improved digital health access not only for everyone but also especially for those in need.

## Methods

### Study Design

We conducted a sequential exploratory mixed methods study to explore a complex phenomenon in depth and then measure its prevalence. We qualitatively explored the experience of digital health for patients with chronic disease and socioeconomic disadvantage in the first phase. Data from the first phase informed a quantitative survey of the phenomenon across a wider population in the second phase [18]. Both stages of research were conducted before the COVID-19 pandemic in Australia.

### Recruitment

#### Qualitative Phase Participants

The eligibility criteria for the qualitative phase were as follows: English-speaking adults aged ≥18 years with at least 1 self-reported chronic disease and 1 marker of socioeconomic disadvantage (indicated by ownership of a Health Care Card or receiving a disability pension, unemployment, or a user of public housing). A chronic disease was defined to potential participants as a diagnosed long-term health condition that had lasted at least 6 months (or is expected to last for at least 6 months; examples are listed in Multimedia Appendix 1). The markers of socioeconomic disadvantage we used to identify potential participants were based on criteria typically used by local general practices to determine which patients can have lower or no out-of-pocket expenses. Apart from unemployment, the 3 other criteria to identify socioeconomic disadvantage are means-tested government-allocated public social services [20]. Qualitative phase participants were recruited from May to July 2019 through 3 general practices and 1 service organization that serve populations considered socioeconomically disadvantaged across urban, regional, and rural regions in the Australian Capital Territory and South Eastern New South Wales. A total of 2 recruitment methods were used in consultation with and as per the choice of the participating organizations. Potential participants were either provided with an opportunity to engage with researchers (KB and MAC) in the general practice waiting room or identified by the practice or organization as suitable for an interview. Interested participants were given a detailed verbal and written description of the project in a private space before providing written consent to be interviewed. All interview participants received an Aus $50 (US $32.68) grocery shopping voucher in acknowledgment of their time.

#### Quantitative Phase Participants

Eligibility for the quantitative phase was English-speaking adults aged ≥18 years. The eligibility criteria for the quantitative phase were deliberately broader than those for the qualitative phase to achieve a larger sample size within the limitations of recruitment and with the intention that the factors of socioeconomic disadvantage and having a chronic disease could be compared to the digital health access of a more general population. The quantitative phase participants were recruited from November 2019 to February 2020. Study information and paper-based surveys were distributed and collected through 24 general practices across the Australian Capital Territory and South Eastern New South Wales regions, with an option for web-based completion.

### Ethical Considerations

Qualitative and quantitative phase research protocols, including the participant information sheet, were approved by the Australian Capital Territory Health Human Research Ethics Committee (2019/ETH/00013) and the Australian National University Human Research Ethics Committee (2019/ETH00003). Qualitative phase participants were given a verbal and written explanation of the study, including how and when they could opt out, before they provided written consent. All interview participants received an Aus $50 (US $32.68) grocery shopping voucher in acknowledgment of their time. Quantitative participants were given a written explanation and their informed consent was implied by return of a completed survey. Participants in both phases of the study were told that all their data was deidentified. Consent was implied through the return of a completed survey.

### Qualitative Data Collection and Analysis

Participants were purposively sampled to represent a range in age, gender, degree of socioeconomic disadvantage, and experience of digital health. The sampling and sample size were reviewed regularly by the research team as the interviews were being completed to identify potential thematic saturation.

The interview guide was developed by the research team based on a review of the literature and the patient dimensions of the framework of access by Levesque et al [21]. The framework by Levesque et al [21] is a conceptualization of health care access comprising 5 service and patient dimensions of accessibility and ability. The patient dimensions are as follows: (1) ability to perceive, (2) ability to seek, (3) ability to reach, (4) ability to pay, and (5) ability to engage [21]. The key interview topics included (1) digital health use and access, including facilitators and barriers; (2) attitudes toward digital health; and (3) self-perception of digital health skills and potential training. The interview guide was reviewed for face and content validity by the whole research team, a patient advocate, a digital inclusion charity representative, and the general practices where recruitment occurred. The questions and guide were iteratively refined by the research team to ensure relevance and support reaching data saturation. The interview guide has been provided as Multimedia Appendix 1. The interviews, which took 45 minutes on average, were taped and transcribed. An interview summary sheet and reflective journal were completed by the interviewer after each interview to also capture nonverbal cues and tone.

Interview transcriptions were coded and processed by inductive thematic analysis. Data collection and analysis were completed in parallel to support the identification of data saturation. Data saturation was defined as no significant new information arising from new interviews and was identified by discussion with the research team [22]. The 2 interviewers (MAC and KB) independently coded the first 5 transcripts and reflected on them with another researcher (EAS) to ensure intercoder validity and reliability. The rest of the interviews were coded independently by the 2 interviewers, who regularly met to reflect on emerging themes and thematic saturation. Data saturation was initially indicated after 15 interviews and subsequently confirmed with a total of 19 interviews. Coding disagreements and theme development were discussed with at least 1 other researcher (EAS, ER, and KD). Thematic saturation and the final themes were agreed upon by the entire research team.

### Quantitative Survey Development

**Figure 1:**
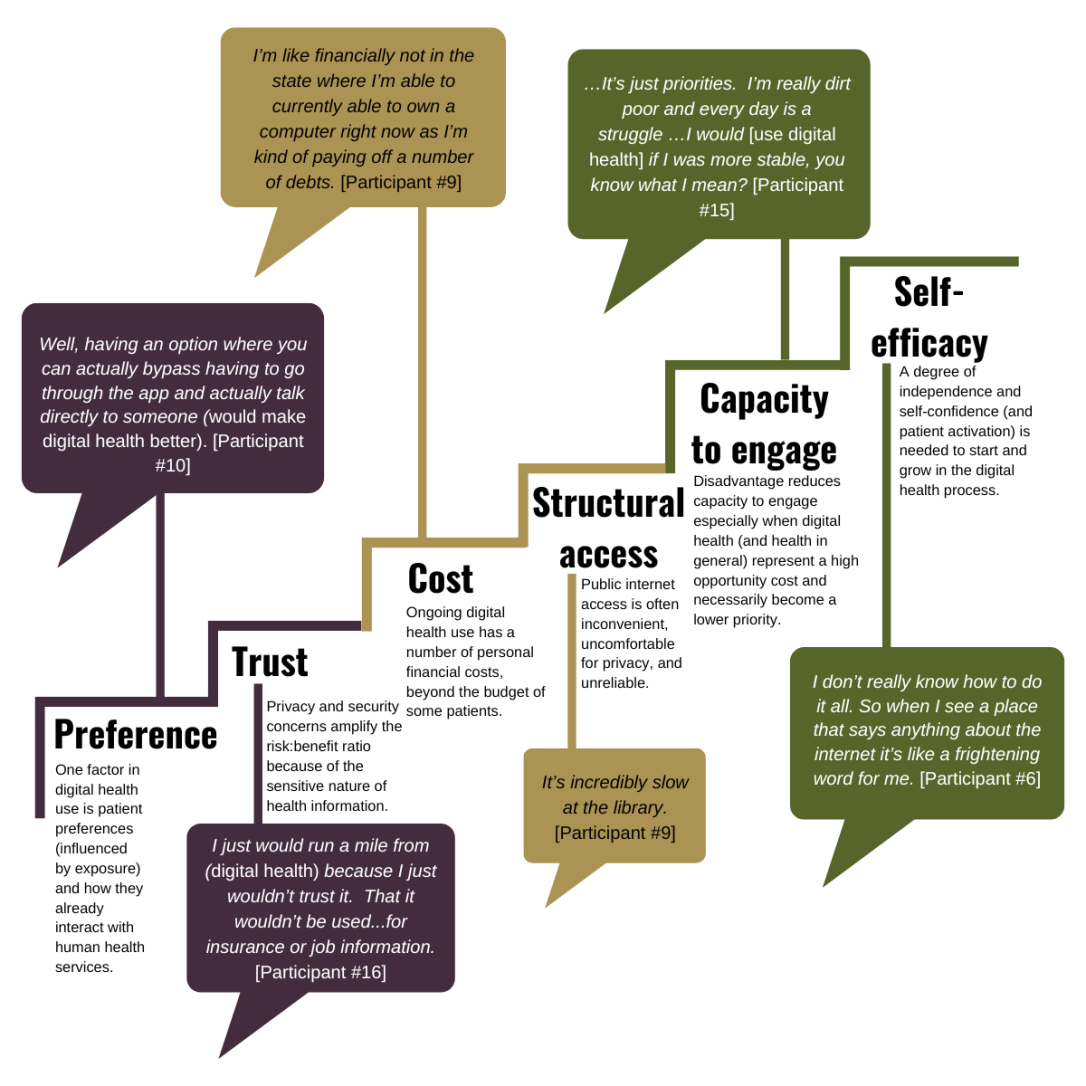
Barriers to digital health access learned in phase 1.

The final themes derived in the qualitative phase of the project guided the specific quantitative phase research questions. The final themes were a list of ordered cumulative barriers experienced by participants in accessing digital health and its benefits (Figure 1). The quantitative survey was designed to test the association between barriers to access and the frequency of use of digital health as a proxy measure for digital health access.

In the survey, the participants were asked about their demographic details, health and chronic diseases, knowledge, use and experience of digital health tools, internet access, perception of digital resource affordability, trust in digital health and traditional health services, perceived capability, health care empowerment, eHealth literacy, and relationship with their GP.

Existing scales and questions from the literature and standardized Australian-based surveys were used whenever possible. We used selected questions and scales from the Australian Bureau of Statistics standards, the eHealth Literacy Scale (eHEALS), the eHealth Literacy Questionnaire, and the Southgate Institute for Health Society and Equity [17,23-26]. We adapted other scales from the ICEpop Capability Measure for Adults, the Health Care Empowerment Inventory (HCEI), the Patient-Doctor Relationship Questionnaire, and the Chao continuity questionnaire [23,27-29]. Where an existing scale to measure a barrier or theme did not exist, the research team designed the questions based on the literature. Our questions around the frequency of digital health use were informed by multiple existing Australian-based surveys on general technology use [30,31]. Most of the questions used a Likert scale. Every choice regarding the design, adaptation, or copy of questions for the survey was influenced by the qualitative findings and decided on by full agreement among the 2 researchers who completed and coded the interviews. A complete copy of the survey is provided in Multimedia Appendix 2.

Pilot-testing of the survey was completed with 5 patients, 2 experts on digital inclusion, and 3 local GPs for both the paper surveys and web-based surveys via Qualtrics Core XM (Qualtrics LLC). The resulting feedback on face and content validity, functionality of the survey logic, and feasibility of questionnaire completion was incorporated into the final version of the survey.

The survey was offered on paper with a participant information sheet, which gave the patients the option to complete the web-based survey. The survey was handed out to every patient on paper to avoid sampling bias through the exclusion of participants who could not complete the web-based survey [32].

### Quantitative Data Treatment and Analysis

Data were exported from Qualtrics Core XM to an SPSS (version 26; IBM Corp) data set. Data cleaning and screening were undertaken (KB and KO).

Descriptive statistics (number and percentage) were used to summarize participant characteristics, preference measures, and frequency of eHealth use. Significance testing was conducted using chi-square tests, with a threshold of *P*<.05; effect sizes were measured by the φ coefficient for 2×2 comparisons and Cramer V statistic for all others. Where the cells sizes were too small, the categories were collapsed for the purposes of significance testing. The interpretation of effect sizes was as per the study by Cohen [33]. The analysis was conducted in SPSS and SAS (version 9.4; SAS Institute).

### Participant Characteristics

Participants’ self-reported characteristics included gender, indigenous status, income category, highest level of education, marital status, and language spoken at home.

Age was derived from participant-reported year of birth and year of survey completion as of 2019 and stratified into age groups. The state or territory of residence was derived from the participant-reported postcode. The remoteness area was derived using the postcode reported by the participants and mapped to a modified concordance from the Australian Bureau of Statistics. Occupation-free text responses were coded using the Australian Bureau of Statistics Census statistics level 1 and 2 descriptors. The country of birth was mapped to Australia, other Organisation for Economic Cooperation and Development countries, and non–Organisation for Economic Cooperation and Development countries.

### Frequency of eHealth Use

A summary measure of the frequency of eHealth use was derived from the questions on the use of different types of eHealth.

Specifically, respondents were asked if they had ever used any form of web-based health (“eHealth“) and, if so, to rate how often (never, at least once, every now and then, and most days) against 6 types of “eHealth” (searching for health information online, booking appointments online, emailing health care providers, using health-related mobile phone apps, accessing My Health Record, and accessing online health forums). The frequency of eHealth use was then classified as follows:

- High user: answered “most days” to at least 1 question on eHealth use OR answered “every now and then” to at least 2 questions on eHealth use
- Never user: answered “no” to having ever used any form of eHealth OR “never” to all 6 questions on eHealth use
- Low or medium user: all other respondents.

The frequency of eHealth use was reported as unweighted descriptive statistics (counts and percentages) against demographic characteristics and for the elements of each of the themes identified in phase 1.

### Themes

#### Overview of Key Themes

Data were reported against the 6 themes from the phase 1 results of preference, trust, cost, structural access, capacity to engage, and self-efficacy. Where the components of trust, cost, capacity to engage, and self-efficacy had missing data (for less than half of the components only), mean imputation was used to minimize data loss. For each theme, the analysis excluded those for whom the frequency of eHealth use was unknown.

#### Preference

Preference measures (survey section D1 parts 1 to 3) asked participants to report against measures with a 4-point Likert scale (strongly disagree, disagree, agree, and strongly agree). Chi-square tests were conducted after the categories were condensed into 2 by combining strongly disagree and as well as combining strongly agree and agree.

#### Trust

Summary measures for trust were created in 4 domains: trust from the eHealth Literacy Questionnaire (survey section D1 parts 4 to 8), trust from Southgate—GPs, specialists, or allied health (survey section D2 parts 1 to 5), trust from Southgate—digital health (survey section D2 parts 6, 7, 9, and 10), and trust from Southgate—books or pamphlets (survey section D2 part 8). The data were grouped as low, moderate, and high trust based on the assigned scores from the component data. Chi-square tests were conducted comparing low-to-moderate trust against high trust for GP, specialists, or allied health and comparing low trust against moderate-to-high trust for book or pamphlet.

#### Cost

Summary measures for cost were created from survey item C10. To measure cost, participants were asked about whether they considered certain items or services to be affordable. These included cost items mentioned in the qualitative phase interviews relating to mobile phones (1 that connects to the internet, 1 with enough memory space to download apps, downloads or apps requiring payment, repairs, and maintenance costs), having an iPad or tablet with internet connectivity, a home computer or laptop (owning, repairs, and maintenance), home fixed internet access, and an adequate monthly data allowance. These 9 items were scored as “yes definitely”=1 or 0 otherwise. Chi-square tests were conducted with never and low or medium eHealth users combined.

#### Structural Access

Structural access included asking where the internet is used by participants (survey section C8) and factors relating to internet access (survey section C8 parts 1-3) reporting against a 4-point Likert scale (strongly disagree, disagree, agree, and strongly agree). Chi-square tests were conducted with strongly disagree, disagree, agree, or strongly agree, and never, low, or medium eHealth use combined.

#### Capacity to Engage

Summary measures for capacity to engage were created from survey section E1. To measure the capacity to engage, participants were asked about feeling “settled and secure,” “being independent,” and “achievement and progress” as an adaptation of the ICEpop Capability Measure for Adults [27], reporting against a 4-point Likert-like scale. Responses were scored from 1 (“I am unable to feel settled and secure in any areas of my life”) to 4 (“I am able to feel settled and secure in all areas of my life”).

The summary capacity measure was derived by the summation of responses across the 3 questions, which were classified into 4 groups, A to D, based on these scores. Where fewer than half of the responses were missing, mean imputation was used; otherwise, the record was excluded. Groups A and B were combined for significance testing.

#### Self-Efficacy

Summary measures for self-efficacy were adapted from the eHEALS (E3) and the HCEI (E2) [23,24].

Survey section E3—eHEALS—comprised 8 questions, with participants reporting against a 5-point Likert scale for each (strongly disagree, disagree, neither, agree, and strongly agree). These responses were assigned 1 to 5 points, respectively. The summary eHEALS measure was derived by the summation of responses across the 8 questions, which were classified into 5 groups, A to E, based on these scores. Where fewer than half of the responses were missing, mean imputation was used; otherwise, the record was excluded. Groups A to C and D to E were combined for significance testing.

Survey section E2—HCEI—comprised 5 questions, with participants reporting against a 5-point Likert scale for each (strongly disagree, disagree, neither, agree, and strongly agree). Strongly disagree and disagree and neither were combined, and similarly agree and strongly agree were combined for significance testing.

## Results

### Qualitative Results

#### Overview

The demographic characteristics of the patients that we interviewed are presented in Table 1.

The key barriers found to accessing digital health included (1) strong patient preference for human-based health services; (2) low trust in digital health services; (3) high financial costs of necessary tools, maintenance, and repairs; (4) poor publicly available internet access options; (5) reduced capacity to engage due to increased life pressures; and (6) low self-efficacy and confidence in using digital health.

Rather than being an equal list of factors, our interviewees described these barriers as a stepped series of cumulative hurdles, which is illustrated in Figure 1. Initial issues of preference and trust were foundational to a person even when considering the option of digital health, while digital health confidence and literacy were barriers to full engagement with and optimal use of digital health. Alternatively, interviewees who did use digital health had been enabled by the same factors that were barriers to others.

**Table 1:** Phase 1 participant characteristics (N=19).

Characteristic			Value, n (%)
**Gender**			
	Female	10 (53)	
	Male	9 (47)	
**Age (years)**			
	18-24	0 (0)	
	25-44	2 (11)	
	45-64	13 (68)	
	65-74	3 (16)	
	>75	1 (5)	
**Country of birth**			
	Australia	13 (68)	
	Other	6 (32)	
**Marital status**			
	Never married	6 (32)	
	Married	4 (21)	
	Widowed	2 (11)	
	Separated, not divorced	2 (11)	
	Divorced	4 (21)	
	Prefer not to say	1 (5)	
**Highest educational qualification**			
	Year ≤11	6 (32)	
	Year 12	4 (21)	
	Trade certification	4 (21)	
	Diploma	3 (16)	
	Bachelor’s degree	1 (5)	
	Prefer not to say	1 (5)	
**Occupation**			
	Not employed	8 (42)	
	Home duties	2 (11)	
	Pension	5 (26)	
	Retired	3 (16)	
	Other	1 (5)	
**Presence of chronic disease**			
	Yes	19 (100)	
	No	0 (0)	
**Number of appointments with GP^a^/year**			
	<5	5 (26)	
	5-10	6 (32)	
	>10	8 (42)	
**Eligibility criteria for socioeconomic disadvantage**			
	User of public housing	6^b^ (32)	
	Disability support pension	10^b^ (53)	
	Health care card	8^b^ (42)	
	Unemployed	3^b^ (16)	

^a^GP: general practitioner.

^b^Multiple answers per respondent.

#### Strong Patient Preference for Human-Based Health Services

Some patients expressed a strong preference for human-based health services rather than digital health services. In answer to a question about how digital health services could be improved, a patient said the following:

Well, having an option where you can actually bypass actually having to go through the app and actually talk directly to someone.

Participant #10

For some patients, this preference for human-based health services appeared to be related to a lack of exposure to eHealth. These patients were not at all interested in or had never thought about digital health options. A participant responded the following to the interviewer’s questions:

Interviewer: So when...something feels not right, how do you find out what’s going on?

Respondent: I talk to Doctor XX.

Interviewer: Do you ever Google your symptoms or look online for information?

Respondent: No, I have never even thought of doing that actually.

Participant #11

For other patients, their preference for human-based health care stemmed from negative experiences with technology. These patients reported actively disliking computers and technology in general and were generally frustrated with what they saw as the pitfalls of technology. A patient stated the following:

If computers and internet weren’t so frigging slow because everything is on like the slowest speed network ever and there’s ads blocking everything. Ads, (expletive) ads.

Participant #9

A patient felt that he was pushed out of the workforce due his inability to keep up with technology-based changes and thus made a decision to never own a computer:

But, you know, in those days when I was a lot younger those sorts of things weren’t about and they’re just going ahead in leaps and bounds and that’s one of the reasons why I retired early. I retired at 63 because it was just moving too fast and it’s all computers and all those sorts of things and I just couldn’t keep up.

Participant #17

#### Low Trust in Digital Health Services

Several patients described low trust levels for digital and internet-based technology in general. Their low trust was generally based on stories they had heard of other people’s negative experiences. A patient said the following:

I don’t trust the internet to be quite honest. You hear all these stories about people getting ripped off and I’ve worked too hard to get what I’ve got rather than let some clown get it on the internet for me.

Participant #11

Some of this distrust was specific to eHealth. For example, some patients were highly suspicious of the government’s motives with regard to digital health and were concerned about the privacy of their health information, which made them hesitant about the concept of a universal electronic health record. In response to the interviewer’s question, a participant said the following:

Interviewer: Are there any other ways you think that eHealth might help you?

Respondent: I’m sorry but it just keeps coming back to me, Big Brother.

Participant #7

Another participant said the following:

I just would run a mile from it because I just wouldn’t trust it. It wouldn’t be used to, as I said, for insurance or job information.

Participant #16

#### High Financial Costs of the Necessary Tools, Maintenance, and Repairs

A wide variety of patients described affordability issues across several different aspects of the costs involved in digital health. They expressed difficulty in paying for the following items: a mobile phone that could connect to the internet, a mobile phone with enough memory space to download apps, mobile phone apps requiring extra payment without advertisements, mobile phone repair costs such as a broken screen, a computer or laptop, home internet access, and adequate monthly data allowance and speeds to functionally use the internet. Current popular payment systems, such as plans, were not feasible for some patients. A participant stated the following:

I don’t have a computer...I’m not in the income bracket to own a computer really. Like I could, if I got one on a plan kind of thing or if I saved up for x-amount of time. But then like if I was going on the plan I’d be paying interest for having it on like lay-buy kind of thing, paying it off, and if it ever got lost or stolen I would still have to repay that off, which is always a hassle. And yeah. Yeah, I’m like financially not in the state where I’m able to...own a computer right now as I’m kind of paying off a number of debts.

Participant #9

#### Poor Publicly Available Internet Access Options

Some patients described struggling without home internet access. While they noted some cost-free public internet access points, such as libraries, hotel bars, and restaurants, they often found these to be inconvenient, lacking in privacy, and constituting low-quality options for digital health. A patient stated the following:

...it’s incredibly slow at the library. And I know why...a friend I went to school with used to belong to the council and the way they set it up, they just got the raw end of the stick and it is really, really slow. It’s bizarre but you can go to the X Hotel and it’s heaps quicker.

Participant #15

In response to the interviewer's question, a participant said the following:

Interviewer: And do you feel comfortable doing private stuff on computers at the library...?

Respondent: Not really, no, but I don’t have any other choice, so, yeah.

Participant #9

#### Reduced Capacity to Engage Due to Increased Life Pressures

When discussing why they were not using digital health or why they had stopped using digital health, patients often described significant competing priorities and life pressures that affected their capacity to engage. An unemployed patient mentioned that his time and energy on the internet were focused primarily on finding work and that he barely had time to focus on his health in general, let alone engage in digital health.

Other patients reported that they often felt that their ability to learn about and spend time on digital health was taken up by caring for sick family members, paying basic bills, or learning English. Some patients said that the time they would have spent learning digital skills when they were growing up had been lost to adverse life circumstances such as being in jail:

So we didn’t have computers in the house when I was growing up. And I didn’t know I’ve never...I’ve been in and out of jail for 28 odd years so it sort of takes away from learning from this cause it’s a whole different… it’s a whole different way of using a telephone from a prison.

Participant #11

#### Low Self-Efficacy and Confidence in Starting the Digital Health Process

Some patients had a pervasive self-perception of being slow learners and being unable to use technology. Their stories of being unconfident learners seemed to stem from the fact that they had been told throughout their lives that they were intellectually behind. A patient said the following:

The computer people...wouldn’t take my calls because I’ve always been dumb with that sort of stuff. Like I only found out this later on in life, but I’m actually severely numerically dyslexic. Like I have to triple-check everything with numbers.

Participant #7

Another patient stated the following:

I like went to two English classes like a normal English class with all the kids and then another English class with about seven kids in there because I just couldn’t I don’t know maybe because I spoke another language at home and they sort of like know I was a bit backward.

Participant #6

These patients and others had multiple missing pieces of information that they felt made it harder to engage in digital health compared to “easier” human-based services. A patient said the following:

Yeah I’ve heard of booking online but I just I don’t know I find it easier just to ring up. And I’ll answer an email from a health care provider but I wouldn’t know where to start to look for their email address.

Participant #11

In contrast, the patients who did connect with digital health described themselves as independent question askers and proactive people. Even when they did not know how to use a specific digital health tool, they were confident in attempting to and asking for help when they needed it. A patient said the following:

I’m a “I will find my way through this, no matter how long it takes me” kind of person. So maybe it’s more my personality...If I have to ask for help from somewhere, wherever it is, I will definitely do that.

Participant #3

### Quantitative Results

#### Participant Characteristics

A total of 487 valid survey responses were received from participants across 24 general practices. The participant characteristics are presented in detail in Table S1 in Multimedia Appendix 3.

The mean age of the participants was approximately 50 years (females 48.9, SD 19.4 years; males 52.8, SD 20.0 years), and 68.2% (332/487) of the participants identified as female. Overall, 34.3% (151/439) of respondents reported never using eHealth, and 53.8% (236/439) reported high eHealth use.

There were statistically significant (*P*<.05) differences in the frequency of eHealth use in terms of age group, gender, state, remoteness, highest level of education, employment status, occupation group, marital status, and language spoken at home, with effect sizes being small to medium. Specifically, high eHealth characteristics were associated with younger age, being female, living in an urban area, and being employed.

#### Preference

Table 2 presents the frequency of eHealth use against 3 internet preference questions.

Preference for using the internet and technology in general and for health needs in particular were significantly related to the frequency of eHealth use (*P*<.05 for each), with the effect sizes being small to medium.

**Table 2:** Preference measures.

		Frequency of eHealth use^a^, n (%)					*P* value^b^			Effect size	
		Never	Low or medium	High	Total						
**I am interested in using the internet and technology in general**								<.001			0.30
	Strongly disagree	13 (72.2)	1 (5.6)	4 (22.2)	18 (100)						
	Disagree	17 (70.8)	4 (16.7)	3 (12.5)	24 (100)						
	Agree	64 (37.2)	22 (12.8)	86 (50)	172 (100)						
	Strongly agree	36 (18.9)	22 (11.6)	132 (69.5)	190 (100)						
**I am interested in using the internet and technology for health needs**								<.001			0.40
	Strongly disagree	18 (75)	1 (4.2)	5 (20.8)	24 (100)						
	Disagree	38 (65.5)	8 (13.8)	12 (20.7)	58 (100)						
	Agree	61 (27.9)	27 (12.3)	131 (59.8)	219 (100)						
	Strongly agree	13 (12.6)	13 (12.6)	77 (74.8)	103 (100)						
**It is important for me to be able to access health resources (eg, information) on the internet**								<.001			0.4
	Strongly disagree	14 (87.5)	0 (0)	2 (12.5)	16 (100)						
	Disagree	35 (63.6)	8 (14.5)	12 (21.8)	55 (100)						
	Agree	65 (30.8)	30 (14.2)	116 (55)	211 (100)						
	Strongly agree	15 (12.6)	11 (9.2)	93 (78.2)	119 (100)						

^a^Excludes those for whom frequency of eHealth use is unknown.

^b^Chi-square tests conducted with strongly disagree and disagree combined, and agree and strongly agree combined.

#### Trust

Table 3 presents the frequency of eHealth use against 4 measures of trust.

The degree of trust was not statistically significantly different for the frequency of eHealth use for any of the domains.

**Table 3:** Trust measures.

			Frequency of eHealth use^a^, n (%)									*P* value			Effect size
			Never		Low or medium		High		Total						
**Trust (as per eHLQ)^b,c^**												.15			0.09
	Low trust	16 (43.2)		6 (16.2)		15 (40.5)		37 (100)							
	Moderate trust	56 (27.1)		25 (12.1)		126 (60.9)		207 (100)							
	High trust	54 (35.3)		18 (11.8)		81 (52.9)		153 (100)							
**Trust^d^ (as per Southgate)—general practitioner, specialist, or allied health**												.42^e^			0.07
	Low trust	5 (83.3)		0 (0)		1 (16.7)		6 (100)							
	Moderate trust	11 (23.9)		9 (19.6)		26 (56.5)		46 (100)							
	High trust	118 (33.3)		39 (11)		197 (55.6)		354 (100)							
**Trust^f^ (as per Southgate): digital health**												.49			0.07
	Low trust	37 (30.8)		19 (15.8)		64 (53.3)		120 (100)							
	Moderate trust	53 (29.4)		21 (11.7)		106 (58.9)		180 (100)							
	High trust	33 (35.5)		8 (8.6)		52 (55.9)		93 (100)							
**Trust^g^ (as per Southgate): book or pamphlet**												.18^h^			0.09
	Low trust	40 (38.5)		11 (10.6)		53 (51)		104 (100)							
	Moderate trust	71 (28.3)		35 (13.9)		145 (57.8)		251 (100)							
	High trust	12 (31.6)		2 (5.3)		24 (63.2)		38 (100)							

^a^Excludes those for whom frequency of eHealth use is unknown.

^b^eHLQ: eHealth Literacy Questionnaire.

^c^Derived from survey question D1, parts 4 to 8. Mean imputation used where ≤2 responses were missing. If >2 responses were missing, the records were excluded.

^d^Derived from survey question D2, parts 1 to 5. Mean imputation used where ≤2 responses were missing. If >2 responses were missing, the records were excluded.

^e^Chi-square test conducted comparing low-to-moderate trust against high trust.

^f^Derived from survey question D2, parts 6, 7, 9, and 10. Mean imputation used where ≤2 responses were missing. If >2 responses were missing, the records were excluded.

^g^Derived from survey question D2 part 8.

^h^Chi-square test conducted comparing low trust against moderate-to-high trust.

#### Cost

Affordability of items and services was reported as *No cost difficulty* or *Cost difficulty.* eHealth frequency of use responses were available for 273 participants; among those with *no cost difficulty*, 1% (2/204) were never users, 14.2% (29/204) were low or medium users, and 84.8% (173/204) were high users of eHealth; among those with *cost difficulty*, 1% (1/69) were never users, 26% (18/69) were low or medium users, and 73% (50/69) were high users. There was a statistically significant difference in the presence of cost as a barrier between never and low or medium eHealth users compared to high users (*χ*^2^_1_=5.25; *P*=.02), although the effect size was small.

#### Structural Access

Table 4 presents the frequency of eHealth use for elements of structural access.

Quality of internet access and feeling limited in access to the internet were significantly associated with frequency of eHealth use (*P*<.05), although the effect sizes were small.

**Table 4:** Structural access.

		Frequency of eHealth use^a^, n (%)					*P* value		Effect size
		Never	Low or medium	High	Total				
**Where do you use the internet most?**							N/A^b^		N/A
	Home	2 (0.8)	45 (18.3)	199 (80.9)	199 (100)				
	Work	0 (0)	4 (10.8)	33 (89.2)	33 (100)				
	Library or other public Wi-Fi	1 (100)	0 (0)	0 (0)	1 (100)				
	Family or friend’s house	1 (100)	0 (0)	0 (0)	1 (100)				
**Overall, my quality of internet access is adequate to suit my needs**							.002^c^		0.18
	Strongly disagree	1 (6.7)	6 (40)	8 (53.3)	15 (100)				
	Disagree	0 (0)	6 (30)	14 (70)	20 (100)				
	Agree	2 (1.3)	26 (16.9)	126 (81.8)	154 (100)				
	Strongly agree	1 (1.1)	10 (10.8)	82 (88.2)	93 (100)				
**I am comfortable dealing with health information online in a public place**							.26^c^		0.07
	Strongly disagree	1 (2.8)	9 (25)	26 (72.2)	36 (100)				
	Disagree	0 (0)	14 (18.7)	61 (81.3)	75 (100)				
	Agree	1 (0.8)	22 (18.2)	98 (81)	121 (100)				
	Strongly agree	1 (2.2)	3 (6.7)	41 (91.1)	45 (100)				
**I feel limited in my access to the internet **							.02^c^		−0.13
	Strongly disagree	1 (0.7)	22 (15.2)	122 (84.1)	145 (100)				
	Disagree	1 (1)	18 (17.3)	85 (81.7)	104 (100)				
	Agree	0 (0)	8 (38.1)	13 (61.9)	21 (100)				
	Strongly agree	1 (12.5)	1 (12.5)	6 (75)	8 (100)				

^a^Excludes those for whom frequency of eHealth use is unknown.

^b^N/A: not applicable (cell sizes insufficient for chi-square test).

^c^Chi-square tests conducted with strongly disagree and disagree combined, agree and strongly agree combined, and never and low or medium categories combined.

#### Capacity to Engage

Table 5 presents the frequency of eHealth use against respondents’ capacity to engage.

Capacity to engage was not significantly different for the frequency of eHealth use (*P*=.54). 

**Table 5:** Capacity to engage.

			Frequency of eHealth use^a^, n (%)									*P* value			Effect size
			Never		Low or medium		High		Total						
**Capacity to engage^b^**												.54^c^			0.06
	Group A: 1-3	2 (50)		0 (0)		2 (50)		4 (100)							
	Group B: 3-6	8 (25.8)		7 (22.6)		16 (51.6)		31 (100)							
	Group C: 6-9	54 (32.5)		17 (10.2)		95 (57.2)		166 (100)							
	Group D: 10-12	72 (34.6)		23 (11.1)		113 (54.3)		208 (100)							

^a^Excludes those for whom frequency of eHealth use is unknown.

^b^Derived from survey item E1. Where 1 response was missing, the mean imputation was used. If >1 response was missing, the record was excluded.

^c^Chi-square tests conducted with groups A and B combined.

#### Self-Efficacy

Table 6 presents the frequency of eHealth use for elements of self-efficacy.

Statistically significant results were observed for the relationship between self-efficacy by eHEALS (moderate effect size) and frequency of eHealth use as well as for some of the questions from the HCEI (reliance on health professionals or others to access and explain information; small effect size; *P*<.05).

**Table 6:** Self-efficacy.

			Frequency of eHealth use^a^, n (%)									*P* value			Effect size
			Never		Low or medium		High		Total						
**Self-efficacy by eHEALS^b,c^**												<.001^c^			0.39
	Group A: 1-8	3 (75)		0 (0)		1 (25)		4 (100)							
	Group B: 9-16	15 (71.4)		3 (14.3)		3 (14.3)		21 (100)							
	Group C: 17-24	47 (58.8)		11 (13.8)		22 (27.5)		80 (100)							
	Group D: 25-32	55 (24.2)		25 (11)		147 (64.8)		227 (100)							
	Group E: 33-40	13 (18.1)		8 (11.1)		51 (70.8)		72 (100)							
**I prefer to get as much information as possible about treatment options**												.08^d^			0.11
	Strongly disagree	3 (60)		0 (0)		2 (40)		5 (100)							
	Disagree	3 (50)		0 (0)		3 (50)		6 (100)							
	Neither	10 (41.7)		6 (25)		8 (33.3)		24 (100)							
	Agree	58 (35.4)		21 (12.8)		85 (51.8)		164 (100)							
	Strongly agree	59 (28.8)		21 (10.2)		125 (61)		205 (100)							
**I try to get my health care providers to listen to my preferences for my treatment**												.06^d^			0.12
	Strongly disagree	3 (75)		0 (0)		1 (25)		4 (100)							
	Disagree	2 (40)		1 (20)		2 (40)		5 (100)							
	Neither	21 (34.4)		12 (19.7)		28 (45.9)		61 (100)							
	Agree	66 (35.5)		17 (9.1)		103 (55.4)		186 (100)							
	Strongly agree	40 (27.2)		18 (12.2)		89 (60.5)		147 (100)							
**I am very active in my health care**												.052^d^			0.12
	Strongly disagree	3 (75)		1 (25)		0 (0)		4 (100)							
	Disagree	5 (25)		3 (15)		12 (60)		20 (100)							
	Neither	22 (28.2)		15 (19.2)		41 (52.6)		78 (100)							
	Agree	71 (37.8)		18 (9.6)		99 (52.7)		188 (100)							
	Strongly agree	31 (27.4)		11 (9.7)		71 (62.8)		113 (100)							
**I take my commitment to my treatment seriously**												.30^d^			0.08
	Strongly disagree	4 (80)		1 (20)		0 (0)		5 (100)							
	Disagree	0 (0)		0 (0)		3 (100)		3 (100)							
	Neither	8 (23.5)		7 (20.6)		19 (55.9)		34 (100)							
	Agree	69 (33.8)		24 (11.8)		111 (54.4)		204 (100)							
	Strongly agree	52 (32.5)		16 (10)		92 (57.5)		160 (100)							
**I rely on health professionals or others to access the information for me and then explain it to me**												<.001^d^			0.22
	Strongly disagree	3 (33.3)		2 (22.2)		4 (44.4)		9 (100)							
	Disagree	3 (7.3)		4 (9.8)		34 (82.9)		41 (100)							
	Neither	18 (21.7)		11 (13.3)		54 (65.1)		83 (100)							
	Agree	68 (37.6)		22 (12.2)		91 (50.3)		181 (100)							
	Strongly agree	41 (45.1)		9 (9.9)		41 (45.1)		91 (100)							

^a^Excludes those for whom frequency of eHealth use is unknown.

^b^eHEALS: eHealth Literacy Scale.

^c^eHEALS derived from item E3 (8 parts). Where ≤ 4 responses were missing, mean imputation was used. If >4 responses were missing, the records were excluded. Groups A to C as well as groups D to E were combined for the chi-square test.

^d^Strongly disagree, disagree, neither, and agree or strongly agree combined for significance testing.

## Discussion

### Principal Findings

This paper reports on the findings of a sequential exploratory mixed methods study on the barriers to digital health access for a group of patients in Australian family medicine, with a particular focus on chronic disease and socioeconomic disadvantage.

In the qualitative first phase, the patients with socioeconomic disadvantage and chronic disease described 6 cumulative barriers, as demonstrated in Figure 1. Many nonusers of digital health preferred human-based services and were not interested in technology, while others were highly suspicious of the technology in general. Some digitally interested patients could not afford quality hardware and internet connectivity, a barrier that was doubled by low quality and privacy when accessing publicly available internet connections. Furthermore, although some digitally interested patients had internet access, their urgent life circumstances left scarce opportunity to access digital health and develop digital health skills and confidence.

In our quantitative second phase, 31% (151/487) of the survey participants from Australian general practices were found to have never used a form of digital health. Survey participants were more likely to use digital health tools frequently when they also had a general digital interest and a digital health interest. Those who did not frequently access digital health were more likely to report difficulty affording the financial costs needed for digital access. The survey participants who frequently accessed digital health were more likely to have high eHealth literacy and high levels of patient empowerment.

### Comparison With Prior Work

In terms of general digital health access, the finding that 31% (151/487) of the survey participants had never used one of the described forms of eHealth is in keeping with an Australian-based general digital participation study that found that approximately 9% of the participants were nonusers and 17% rarely engaged with the internet at all [34]. With regard to the digital health divide, another Australian-based digital health divide study found that increased age, living in a lower socioeconomic area, being Aboriginal or Torres Strait Islander, being male, and having no tertiary education were factors negatively associated with access to digital health services [17]. Their findings correspond to our findings that higher-frequency users of eHealth were associated with younger age, being female, living in an urban area, and being employed. Both studies reinforce the evidence of the digital health divide based on gender, age, and socioeconomic disadvantage in Australia.

With regard to digital health barriers, our findings provide expanded details on the range of digital health items and services that present a cost barrier to consumers. Affordability is a known factor in digital access and digital health access, and it is measured often by general self-report or relative expenditure on internet access to income [30]. Our study revealed the comprehensive list of relevant costs for patients. Our study also demonstrated factors of cost affordability beyond the dollar value of an item, as interviewees described the struggle of using slow public internet access without privacy features and the risks involved in buying a computer in installments. When we reflected on the complexity and detail of the cost barrier in our survey, participants demonstrated a clear association between cost and the frequency of digital health use. This suggests that a way to improve digital health access for some people is to improve the quality, security, and accessibility of public internet access options as well as to provide free or subsidized hardware, internet connection, and maintenance options for those in need, work that is being done by at least 1 digital inclusion charity in the United Kingdom [35].

Many studies recognize the factors of eHealth literacy and digital confidence for beneficial digital health access [36]. Our interviews demonstrated that some patients with socioeconomic disadvantage have low digital confidence, but that this is often underlined by a socially reinforced lifelong low self-confidence in their intellectual ability. In contrast, active users, regardless of other demographic factors, described themselves as innately proactive question askers. This was reinforced by our finding of a relationship between health care empowerment and the frequency of eHealth use. This suggests that while digital health education and eHealth literacy programs can improve access for some patients, broader and deeper long-term solutions addressing socioeconomic drivers of digital exclusion are needed to improve digital health access for some patients with socioeconomic disadvantage [8]. The deep permeation of socially enforced low self-confidence and lifelong poverty experienced by some interviewees demonstrate that the provision of free hardware and a class on digital health skills can be, for some, a superficial offering when the key underlying factor is persistent general socioeconomic inequality.

The digital health divide literature tends to identify the digital health divide, the factors and barriers that contribute to it, and the potential for it to widen if not specifically addressed [16]. Our findings have also identified the divide and the barriers, but what this study adds through our qualitative phase in particular is a description of the complex interaction of those barriers and the stepped nature of some of those barriers as part of the individual’s experience in trying to access digital health.

### Strengths and Limitations

A key strength of this study is the use of a sequential exploratory mixed methods design. The initial qualitative phase guided a phenomenological exploration of digital health access experiences for patients with chronic disease and socioeconomic disadvantage. Our results in both study phases stem from the patients’ real-life experiences of digital health access. While some of our results echo the findings of other survey-based studies on general digital and digital health participation, our method revealed a greater depth and detail of some of these barriers, as demonstrated in how our findings compare to prior work.

As mentioned previously, the emphasis of this study on the qualitative first phase is a strength that helped describe the interactions between different barriers. The interviewees described their experiences as cumulative unequal stepped barriers rather than as producing a nonordered list of equal barriers. These findings expand on the known complexity of the issue of digital exclusion and add weight to the understanding that improving digital health access needs diverse, complex solutions [17]. There is no panacea for every individual’s digital health access, and thus, patient-centered digital health services, often guided by health professionals within the continuity of primary care, are also required to address the digital health divide [37].

While the sequential exploratory design is a strength of the study, it also created some limitations for the second quantitative phase. Our commitment to using the qualitative interview findings to inform the survey questions meant that we were unable to use previously validated scales for every question and that our results were less likely to lead to a normal distribution. This likely affected our ability to demonstrate significant associations for some barriers. We expect that further modeling is required to control for baseline characteristics and determine barrier patterns for different types of users.

One strength of this study is that the survey was administered to a broad population of Australian family medicine patients with diverse patterns of health via both paper-based and digital options. Many other digital health studies use solely digital surveys, which can affect the sample. However, we cannot draw conclusions from our survey about patients with chronic disease due to the limitations of the sample size for these subgroups.

Another sample-based limitation of this study was that our qualitative population did not include anyone aged from 18 to 24 years, despite multiple efforts to recruit. Future research will hopefully address this demographic more specifically.

While not strictly a limitation, we recognize that because this research was before COVID-19, it did not include questions about telehealth, which has become much more mainstream in recent years. The patients may also have changed their frequency of eHealth use because of COVID-19 and an increased reliance on digital services in general. Future work in this area or future versions of this survey should include telehealth and acknowledge the impact of COVID-19. However, the larger concept of the digital health divide exists before and after COVID-19, and in fact, our widespread increased reliance on digital services makes the digital divide an even more pressing issue [12].

### Conclusions

The experience of digital health access across Australian primary care is highly variable and more difficult to access for those with socioeconomic disadvantage. While general digital interest, financial cost, and digital health literacy and empowerment are clear factors in digital health access in a broad primary care population, the digital health divide is also facilitated in part by a stepped series of complex and cumulative barriers.

Genuinely improving digital health access for 1 cohort or even 1 person requires a series of multiple different interventions tailored to specific sequential barriers. Given the rapid expansion of digital health during the global COVID-19 pandemic, attention to these issues is necessary if we are to avoid entrenching inequities in access to health care. Within primary care, patient-centered care that continues to recognize the complex individual needs of, and barriers facing, each patient should be a part of addressing the digital health divide.

## Appendix

Multimedia Appendix 1 Phase 1 interview guide.

Multimedia Appendix 2 Phase 2 survey: eHealth and digital divide.

Multimedia Appendix 3 Phase 2 participant characteristics by frequency of eHealth use.

## Abbreviations

eHEALS: eHealth Literacy Scale
GP: general practitioner
HCEI: Health Care Empowerment Inventory

## Data Availability

The data sets generated during this study are not publicly available due to the nature of our original ethics approval but are available from the corresponding author on reasonable request.
