# Supplementary material for: Evaluating the Digital Health Experience for Patients in Primary Care: Mixed Methods Study
Source: J Med Internet Res. 2024 Apr 11;26:e50410. doi: 10.2196/50410 (PMC11046385; doi:10.2196/50410)
Supplement: Multimedia Appendix 1 [file jmir_v26i1e50410_app1.docx]

This is a Multimedia Appendix to a full manuscript published in the J Med Internet Res. For full copyright and citation information see http://dx.doi.org/10.2196/jmir.50410.

**Research questions**

- How do patients with socioeconomic disadvantage view and experience ehealth in the context of their chronic disease?
  - Specifically, what types of positive and negative attitudes do they report?
- What are the correlates (and potential determinants) of engagement with ehealth and positive/negative attitudes towards ehealth?
- Would ehealth skills training be positively received by patients with chronic disease and socioeconomic disadvantage? And, if so,
- How could ehealth skills training be best designed for uptake and engagement?

**Demographic questionnaire (see Appendix A for full version)**

- Age (in age brackets)
- Sex
- Country of birth
- Marital status
- Occupation, if any
- Highest educational qualifications
- Household income (in household income brackets)
- A chronic disease is a long term health condition.
  - Examples include arthritis, long-term back pain, cancer, coronary heart disease, stroke, chronic obstructive pulmonary disease, diabetes and mental health conditions (such as depression).
  - You may have a long term health condition if you have ever been told by a doctor or nurse that you have a particular health condition, that the condition is current today, and the condition has lasted at least six months or more, or you expect it to last six months’ or more.
  - Do you have a long-term health condition?
  - How many long-term health conditions do you have?

**Interview guide**

Introduction

This interview is about your experience of ehealth. We would define ehealth as the use of technology for health purposes. Examples of this can include searching for health information online, booking appointments online, email communication with healthcare providers, using health related phone apps, My Health Record, other government or non-government health-based websites.

(Show **infographic** to the patient)

Access to and information about ehealth

| **Question** | | **Follow up questions** | | **Aims** |
| --- | --- | --- | --- | --- |
| In general, what do you think about **technology**/digital things? |  | Why is that? |  | Exploring general attitudes towards digital technology. |
| (Do you access the Internet?)  How do you normally **access** and use the Internet? |  | (If no)  Does anyone access the Internet for you or with you? |  | Identifying type of Internet use and type of Internet user. |
|  |  | What other types of access do you have?  Mobile?  Home?  Public space? (Libraries)  How comfortable are you with accessing the internet for different services in these locations? |  |  |
|  |  | Where are you physically when you access the Internet? |  |  |
|  |  | What services do you use the Internet for? eg banking, hair appointments, games, social media, entertainment, work etc |  |  |
| How **reliable** is your Internet access? |  | Does anything restrict your ability to use the Internet? |  | Exploring potential material, cost and infrastructure barriers to Internet access, and ehealth use. |
|  |  | How expensive is it to use? (data etc) |  |  |
|  |  | Have you ever lost access to the Internet in a way that affected you? What happened? |  |  |
|  |  | Any issues with hardware? (computer, phone) What impact does that have? |  |  |
|  |  | How does your level of Internet access affect your ability to use ehealth? |  |  |
| How **aware** of ehealth were you before today? |  | Did you know that you could use the different types of ehealth on the infographic to help you manage your health? |  | Assessing general ehealth awareness. |
|  |  | How much did you know about these things? |  |  |
| How would you go about **answering a question** that you might have about your health?  (can give example of a relevant question) |  | Can you give me an example of that?  How would you do it online?  How useful is that information to you? How much do you trust that information?  What websites have you used before for health information?  Have you ever looked up something that you were too uncomfortable or embarrassed to speak to your doctor about? (don’t need details of what) |  | Assessing whether they know where to find, how to use the internet, how to assess resources, confidence, features of ehealth literacy. |
| Do you know if **your doctor** or your general practice uses or talks about ehealth? |  | Does your practice use any ehealth tools? eg appointments online or electronic registration? How do you feel about this? |  | Exploring how GPs affect patient engagement with ehealth and whether they are a source of information. |
|  |  | Has your doctor ever given you any information on ehealth or ehealth tools eg infographic? Has your doctor asked you to use a health app or look something up online about your health? Did you do it? Why/why not? |  |  |
|  |  | Would you like your doctor to use or discuss ehealth with you more? |  |  |

Ehealth tool use patterns

| **Question** | | **Follow up questions** | **Aims** | |
| --- | --- | --- | --- | --- |
| Can you tell me about how you **manage your health**? |  | What are some of the hardest things about living with a chronic disease? |  | Understanding how health beliefs and perceived health literacy affect ehealth use.  Contextualising health access in general. |
|  |  | How well do you understand your chronic disease and how should be managed? |  |  |
|  |  | Tell me about your experiences with health services at the moment. |  |  |
| If at all, how does your **health/ chronic disease affect** your ability to use ehealth tools? |  | How? / In what ways? |  | Exploring effect of chronic disease on ehealth use, relative to general health access. |
|  |  | Does your health/chronic disease impact your ability to use general health services? Which and how? |  |  |
| Have you ever **use**d an ehealth tool? |  | If never used: Why have you not used ehealth tools up until now? 🡪 then skip next question |  | Assess whether any use of ehealth. |
| Can you tell me about **a time when you used** an ehealth tool? |  | Can you tell me about X? |  | Understanding:  - how ehealth is used  - types of ehealth used  - frequency of ehealth use  - how ehealth used  - starting/stopping factors and adherence  - purpose of use |
|  |  | Why did you start using X? or  What did you use X for? |  |  |
|  |  | How often do you use X? |  |  |
|  |  | Why do you continue to use X?, or  Why did you stop using X? |  |  |
|  |  | What about (whatever not covered of the following:) searching for health information online,  booking appointments online,  email communication with healthcare providers,  using health related phone apps,  My Health Record,  Online health forums  Other govt or non govt health based sites? Have you ever used any of these? |  |  |
|  |  | What do you use ehealth tools for mostly? |  |  |
|  |  | Are there any forms of ehealth that you would not consider using?  Why is that? |  |  |
| Do you know whether anyone in your **family or social circle** uses ehealth tools? |  | Who? |  | Exploring family and community pattern of and potential influences on personal ehealth use. |
|  |  | Which ehealth tools do they use? |  |  |
|  |  | How do they feel about ehealth? |  |  |
|  |  | Have they ever recommended any ehealth tools to you? Or told you negative things about ehealth tools? |  |  |
| *(If using any ehealth tools)*  Has an ehealth tool ever **helped** you? |  | Which one?  How? |  | Exploring effect of ehealth use. |
|  |  | Can you tell me about a time that you decided to see a doctor or decided not to see a doctor after using an ehealth tool?  How did that happen? |  |  |
|  |  | Has an ehealth tool ever made your life harder? How? |  |  |

Perspectives on ehealth tools

| **Question** | | **Follow up questions** | | **Aims** |
| --- | --- | --- | --- | --- |
| **What do you think** about ehealth? |  | Why? |  | Gathering general and specific perspectives on ehealth. |
|  |  | Are you interested in ehealth? |  |  |
|  |  | How do you feel when you look at health information online? |  |  |
|  |  | How do you feel about My Health Record? Why? |  |  |
| How do you think ehealth could **help you manage** your health? |  | Can you imagine ways that ehealth might help you? |  | Considering imagined benefits |
|  |  | Why do you think other people use ehealth? What might they be using it for? |  |  |
| What **has helped** or would help you to use ehealth tools more? |  | How did that work? |  | Understanding enablers for ehealth use (potential and actual). |
| What are some **obstacles** to using ehealth for you? |  | How did that work? |  | Understanding barriers for ehealth use. |
| What are your concerns, if any, about the **security** of your information when you use ehealth tools? |  | Have your concerns stopped you from using a particular ehealth tool? |  | Trying to see if trust and security issues are a barrier. |
| When have you found ehealth tools or technology in general **frustrating?** |  | How often? What happens? |  | Understanding how expectations affect ehealth use. |
|  |  | Do you think these experiences stop you from using ehealth tools and technology more? |  |  |
|  |  | What do you hope ehealth can do for you? |  |  |
| What **type of patients** do you think use ehealth the most? |  | Why is that? |  | Asking about how personal, social, cultural values affect ehealth use. |
|  |  | Do you identify as someone who who would benefit from ehealth? Why/why not? |  |  |

Ehealth literacy level and skills training

| **Question** | | **Follow up questions** | | **Aims** |
| --- | --- | --- | --- | --- |
| How **confident** (and skilled) are you in using technology and digital tools eg email, general searching?  How **confident** (and skilled) would you say you are in using ehealth tools? |  | Do you know what technology or ehealth skills you are missing? What effect does that have on you? |  | Exploring self-perception of digital skills |
|  |  | What parts of using ehealth do you find easy? |  |  |
|  |  | What parts of using technology do you find easy? |  |  |
|  |  | What would help you increase your confidence in using ehealth tools? |  |  |
| (If user of ehealth)  How did you **learn** to use [particular ehealth thing they use] (use infographic)?  (Ask about each type specifically) |  | Did anyone teach or help you? Who? |  | Exploring barriers of and avenues to empowerment, information and support. |
|  |  | Do you wish someone had helped or taught you? |  |  |
|  |  | Have you become better at using X over time? |  |  |
| Where would you go to find **help** if you needed it in using ehealth? (or use specific examples of types they don’t use) |  | How did you know about those? |  | Exploring barriers to and enablers of information about how to use ehealth. |
| Would you be **interested in support** in using ehealth tools to manage your health? |  | What sort of support do you think would be most useful to you? |  | Gauging interest in support, nature of support. |
|  |  | Are there any particular things you want to learn how you use? |  |  |
|  |  | What would make it easier or harder for you to benefit from that training? |  |  |
| If **ehealth skills support** were to be offered, what would make you more or less interested in attending it? |  | Under what circumstances would you look for ehealth support? |  | Understanding further opinions and needs in ehealth skills training |
|  |  | Who would you trust to help you with ehealth? |  |  |
|  |  | What is the best way for that information to be delivered? Where would you prefer to go to get support? (eg library) |  |  |
