# Supplementary material for: Evaluating the Digital Health Experience for Patients in Primary Care: Mixed Methods Study
Source: J Med Internet Res. 2024 Apr 11;26:e50410. doi: 10.2196/50410 (PMC11046385; doi:10.2196/50410)
Supplement: Multimedia Appendix 2 [file jmir_v26i1e50410_app2.docx]

This is a Multimedia Appendix to a full manuscript published in the J Med Internet Res. For full copyright and citation information see http://dx.doi.org/10.2196/jmir.50410.

## **Ehealth and the digital divide**

## *Research Project: Phase Two Survey*

**GENERAL DETAILS**

**A2. What is your home postcode?** ____________

**A3. In what year were you born?** ____________

**A4. In what country were you born?** _______________________________

**A5. Do you usually speak a language other than English at home?**

- No
- Yes (please specify) _____________________________________________

**A6. What is your gender?** Please mark one box.

- Female
- Male
- Non-binary
- Prefer to self-describe _____________________________________________
- Prefer not to say

**A7. Are you of Aboriginal or Torres Strait Islander origin?**

For persons of both Aboriginal and Torres Strait Islander origin, mark both ‘Yes’ boxes.

- No
- Yes, Aboriginal
- Yes, Torres Strait Islander
- Prefer not to say

**A8. What is your marital status?**

- Never married
- De facto
- Married
- Separated, but not divorced
- Divorced
- Widowed
- Other ______________________

Please continue over the page.

1

**A9. Which of the following best describes your employment status?**

- Employed (full-time or part-time, including self-employed)
- Not employed, seeking work
- Not employed, not seeking work
- Retired

**A10. What is/was your occupation?**Please give full title. For example: childcare worker, maths teacher, pastry cook, sheep and wheat farmer, apprentice toolmaker. For public servants, provide official designation and occupation (e.g. APS4, health administration officer).

__________________________________________________________________________________________________________________________________________________

- Not currently employed

**A11. What is the highest educational qualification you have completed?** Please mark one box.

- Postgraduate degree /diploma
- Bachelor degree
- Diploma/Advanced diploma
- Certificate III/IV
- Year 12 or equivalent
- Year 11 or equivalent
- Year 10 or below
- Other (please specify) ____________________________
- Did not go to school
- Prefer not to say

**A12. What is your approximate average personal income before tax?**(Include benefits, pensions, superannuation etc.) Please mark one box.

- $2,000 or more per week
  ($104,000 or more per year)
- $1,500 to $1,999 per week
  ($78,000 to $103,999 per year)
- $1,000 to $1,499 per week
  ($52,000 to $77,999 per year)
- $500 to $999 per week
  ($26,000 to $51,999 per year)
- $300 to $499 per week
  ($15,600 to $25,999 per year)
- $150 to $299 per week
  ($7,800 to $15,599 per year)
- Less than $150 per week
  (less than $7,800 per year)
- No regular income
- Prefer not to say

**CHRONIC DISEASES / CONDITIONS**

A chronic condition or disease is a long term health condition that has lasted at least six months (or is expected to last at least six months).

**B1. Do you have any of the chronic conditions listed below?**Please mark all that apply.

- Arthritis
- Bowel disease (e.g. irritable bowel syndrome, ulcerative colitis, Crohn’s disease, diverticular disease)
- Cancer in the preceding 5 years (including melanoma but excluding other skin cancers)
- Chronic hepatitis
- Chronic lung condition (e.g. asthma, COPD, chronic bronchitis)
- Chronic pain condition (e.g. chronic back pain, migraines)
- Dementia or Alzheimer’s disease
- Depression or anxiety
- Diabetes mellitus (type 1 or type 2)
- Heart failure or heart disease (e.g. angina, ischaemic heart disease, peripheral vascular disease)
- High blood pressure
- High cholesterol
- Kidney disease (e.g. kidney failure, chronic urinary tract infections)
- Musculoskeletal condition
- Obesity
- Osteoporosis
- Reflux or gastric ulcer disease
- Stroke or transient ischaemic attack
- Thyroid disease
- Other _____________________
- I do not have any chronic conditions.

**B2. About how many health appointments have you had over the past 12 months?**

| Type of health professional | None | Less than 5 | 5-10 | More than 10 |
| --- | --- | --- | --- | --- |
| General Practitioner (GP) | 🞏 | 🞏 | 🞏 | 🞏 |
| Allied health e.g. physiotherapist, dietitian, psychologist | 🞏 | 🞏 | 🞏 | 🞏 |
| Specialist doctor (non GP) | 🞏 | 🞏 | 🞏 | 🞏 |

**B3. Do you feel your chronic condition(s) is/are under control?**

- Yes
- Somewhat in control
- Not in control
- I do not have a chronic disease or condition.

### **TECHNOLOGY AND EHEALTH USE**

**C1. How often do you use *the internet in general* with the following type of device?** Please mark all that apply. Examples of internet use might be Facebook, email, Google etc.

- I do not use the internet at all.

| Type of device | Never | At least once | Every now and then | Most days |
| --- | --- | --- | --- | --- |
| My own mobile phone | 🞏 | 🞏 | 🞏 | 🞏 |
| Another person’s mobile phone | 🞏 | 🞏 | 🞏 | 🞏 |
| My own iPad/tablet | 🞏 | 🞏 | 🞏 | 🞏 |
| My own computer or laptop | 🞏 | 🞏 | 🞏 | 🞏 |
| A free public computer or laptop e.g. library | 🞏 | 🞏 | 🞏 | 🞏 |
| Another person’s computer/laptop | 🞏 | 🞏 | 🞏 | 🞏 |
| A work computer/laptop | 🞏 | 🞏 | 🞏 | 🞏 |

Technology is being used more and more to help people manage their health. *Ehealth is the use of any type of technology to manage health.* Ehealth can be used by patients, hospitals and healthcare providers. We are interested in *ehealth used by patients*.

**C2. Which of the following examples of ehealth used by patients *are you aware of*?** Please circle all the large coloured boxes that apply, even if you have never used them before.

| 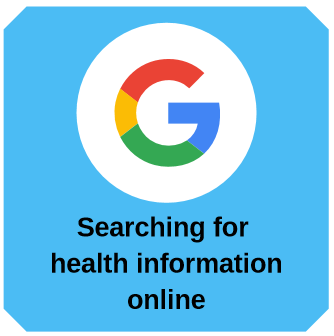 | 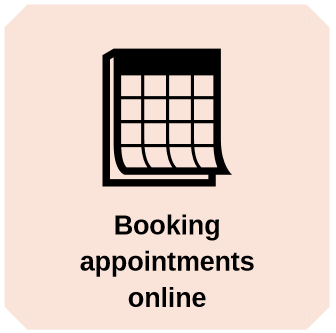 | 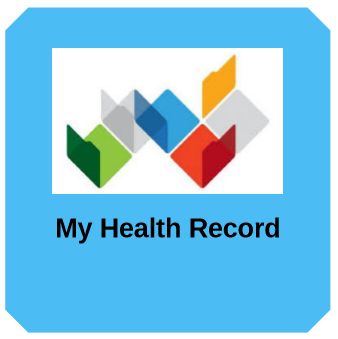 | 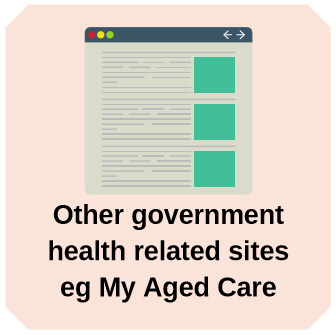 |
| --- | --- | --- | --- |
|  |  |  |  |
| 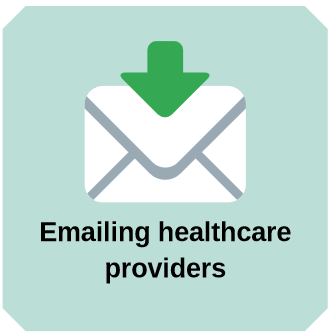 | 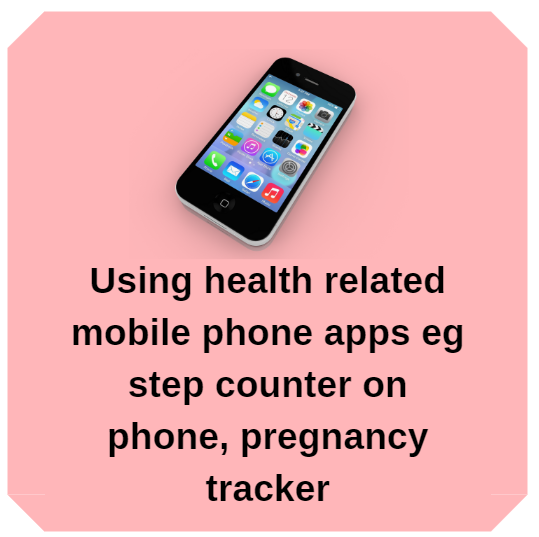 | 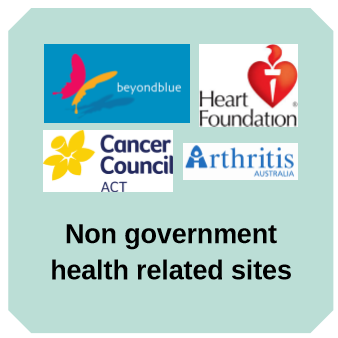 | 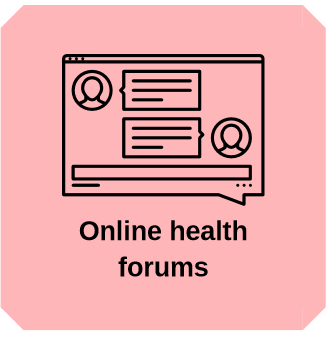 |

**C3. Have you ever used *any form of ehealth*, such as the examples above?** (searching for health information online, booking appointments online, emailing healthcare providers, using health-related mobile phone apps, My Health Record, other government or non-government health sites, online health forums)

- Yes (please continue to the next question C4 over the page)
- No

**If you have answered No:**

Why don’t you use ehealth?

__________________________________________________________________________________________________________________________________________________________________________________________

Is there anything that might help you to use ehealth?

__________________________________________________________________________________________________________________________________________________________________________________________

**If you have answered ‘No’ to the question above please *skip* to the at the end of page 8.**

**large black rectangle**

If you have answered ‘Yes’ to ever using any form of ehealth, please continue answering the following questions.

**C4. How often do you use the following forms of ehealth?** Please mark all that apply. This could apply to yourself or using ehealth on behalf of someone else.

|  | Never | At least once | Every now and then | Most days |
| --- | --- | --- | --- | --- |
| Searching for health related information online e.g. Google | 🞏 | 🞏 | 🞏 | 🞏 |
| Booking appointments online | 🞏 | 🞏 | 🞏 | 🞏 |
| Emailing a healthcare provider | 🞏 | 🞏 | 🞏 | 🞏 |
| Health-related mobile phone app | 🞏 | 🞏 | 🞏 | 🞏 |
| Accessing My Health Record | 🞏 | 🞏 | 🞏 | 🞏 |
| Accessing an online health forum | 🞏 | 🞏 | 🞏 | 🞏 |

**C5. How helpful have you found the following forms of ehealth?**

|  | Not at all helpful | A bit helpful | Helpful | Very helpful | Have not used |
| --- | --- | --- | --- | --- | --- |
| Searching for health related information online e.g. Google | 🞏 | 🞏 | 🞏 | 🞏 | 🞏 |
| Booking appointments online | 🞏 | 🞏 | 🞏 | 🞏 | 🞏 |
| Emailing a healthcare provider | 🞏 | 🞏 | 🞏 | 🞏 | 🞏 |
| Health-related mobile phone app | 🞏 | 🞏 | 🞏 | 🞏 | 🞏 |
| Accessing my own My Health Record | 🞏 | 🞏 | 🞏 | 🞏 | 🞏 |
| Accessing an online health forum | 🞏 | 🞏 | 🞏 | 🞏 | 🞏 |

**C6. Through using ehealth, I have experienced:**

This may be for your health or the health of your family. Please mark all that apply.

- More communication with healthcare professionals
- Connection and support from others when I need it (e.g. health forums on a bad day)
- More health information (e.g. information about medications)
- Increased anxiety about health
- Confusion about health conditions or symptoms
- Feeling worse about my health condition
- Easier to understand health information
- Regular health reminders and monitoring
- An increased ability to manage my health
- Motivation to make good decisions for my health
- Decreased anxiety about health
- Flexibility and convenience in managing my health
- Increased privacy in meeting healthcare needs
- Decreased privacy in meeting healthcare needs
- Increased confidence in healthcare providers and systems
- Other_______________________

**C7. Who has recommended a form of ehealth to you or helped you use an ehealth tool?** Please mark all that apply.

|  | Recommended ehealth to me | Helped me use ehealth tool |
| --- | --- | --- |
| Family member, friend and/or colleague | 🞏 | 🞏 |
| Carer | 🞏 | 🞏 |
| General Practitioner (GP) | 🞏 | 🞏 |
| Specialist doctor (non GP) | 🞏 | 🞏 |
| Allied healthcare professional e.g. dietitian, occupational therapist, psychologist etc. | 🞏 | 🞏 |
| Library or other community organisation staff | 🞏 | 🞏 |
| An advertisement | 🞏 | 🞏 |
| Other________________________________ | 🞏 | 🞏 |
| Other________________________________ | 🞏 | 🞏 |

- I have not had an ehealth tool recommended to me.

**C8. Where do you use the internet the most?** Please mark one box.

- Home
- Work
- Library/other publicly available WiFi
- Family member or friend’s house
- Other _____________________

**C9. Please indicate how much you agree or disagree with each of the following statements.**

|  | Strongly disagree | Disagree | Agree | Strongly agree |
| --- | --- | --- | --- | --- |
| I find that my *quality of internet access* (speed etc.) is adequate to suit my needs | 🞏 | 🞏 | 🞏 | 🞏 |
| I am *comfortable* dealing with health information online *in a public place* | 🞏 | 🞏 | 🞏 | 🞏 |
| I feel *limited* in my access to the internet | 🞏 | 🞏 | 🞏 | 🞏 |

**C10. Are the following items and services affordable for your personal/family budget (even if you don’t use them)?**

|  | Yes, definitely | Yes, with some difficulty | No | Unsure |
| --- | --- | --- | --- | --- |
| *A mobile phone* that connects to the internet | 🞏 | 🞏 | 🞏 | 🞏 |
| *A mobile phone with enough memory space* to download apps | 🞏 | 🞏 | 🞏 | 🞏 |
| *Mobile phone program or app downloads* that require extra payment e.g. ones that don’t have ads | 🞏 | 🞏 | 🞏 | 🞏 |
| *Mobile phone repairs and maintenance costs* | 🞏 | 🞏 | 🞏 | 🞏 |
| *An iPad/tablet* that connects to the internet | 🞏 | 🞏 | 🞏 | 🞏 |
| *A home* computer/laptop | 🞏 | 🞏 | 🞏 | 🞏 |
| *Home* computer/laptop *repairs and maintenance costs* | 🞏 | 🞏 | 🞏 | 🞏 |
| Home *fixed internet access* (e.g. WiFi or cable internet at home) | 🞏 | 🞏 | 🞏 | 🞏 |
| *Adequate monthly data allowance* for my needs | 🞏 | 🞏 | 🞏 | 🞏 |

**Please keep answering all the questions from the next page onwards,**

**whether you have ever used ehealth before or not.**

**The rest of the questions in this survey are for everyone.**

### **OPINIONS ON TECHNOLOGY AND EHEALTH**

The following questions ask what you think about ehealth.

**D1. Please indicate how much you agree or disagree with each of the following statements.**

|  | Strongly disagree | Disagree | Agree | Strongly agree |
| --- | --- | --- | --- | --- |
| I am interested in *using the internet and technology in general* | 🞏 | 🞏 | 🞏 | 🞏 |
| I am interested in *using the internet and technology for health* *needs* | 🞏 | 🞏 | 🞏 | 🞏 |
| It is important for me to be able *to access health resources (e.g. health information) on the internet* | 🞏 | 🞏 | 🞏 | 🞏 |
| I am sure that my health data are *being used only by those who are supposed to use it* | 🞏 | 🞏 | 🞏 | 🞏 |
| My electronic healthcare data are being *stored safely* | 🞏 | 🞏 | 🞏 | 🞏 |
| I have a *clear understanding* of how healthcare providers use my data | 🞏 | 🞏 | 🞏 | 🞏 |
| I am sure that *only authorised people* can access my health data | 🞏 | 🞏 | 🞏 | 🞏 |
| I am confident that healthcare *providers use my data appropriately* | 🞏 | 🞏 | 🞏 | 🞏 |

**D2. How much would you trust the information and advice provided by the following people/places?** (Please answer even if you have never used/seen them.)

|  | Wouldn’t trust it at all | Would only trust it a little bit | Would trust it a reasonable amount | Would trust it complet-ely |
| --- | --- | --- | --- | --- |
| GP | 🞏 | 🞏 | 🞏 | 🞏 |
| Specialist doctor (non GP) | 🞏 | 🞏 | 🞏 | 🞏 |
| Nurse | 🞏 | 🞏 | 🞏 | 🞏 |
| Pharmacist | 🞏 | 🞏 | 🞏 | 🞏 |
| Other allied health e.g. physiotherapist | 🞏 | 🞏 | 🞏 | 🞏 |
| Information that I have found myself on the internet | 🞏 | 🞏 | 🞏 | 🞏 |
| A health-related mobile phone program or application | 🞏 | 🞏 | 🞏 | 🞏 |
| A book or pamphlet about health | 🞏 | 🞏 | 🞏 | 🞏 |
| My Health Record | 🞏 | 🞏 | 🞏 | 🞏 |
| Online health forums with peer support advice | 🞏 | 🞏 | 🞏 | 🞏 |

### **FINAL GENERAL QUESTIONS**

The next few questions will ask about your life in general, and your involvement in your health care.

**E1. Please mark one box per question with what best describes your life currently.**

E1a. Feeling settled and secure

- I am able to feel settled and secure in all areas of my life
- I am able to feel settled and secure in many areas of my life
- I am able to feel settled and secure in a few areas of my life
- I am **unable** to feel settled and secure in any areas of my life

E1b. Being independent

- I am able to be completely independent
- I am able to be independent in many things
- I am able to be independent in a few things
- I am **unable** to be at all independent

E1c. Achievement and progress

- I can achieve and progress in all aspects of my life
- I can achieve and progress in many aspects of my life
- I can achieve and progress in a few aspects of my life
- I **cannot** achieve and progress in any aspects of my life

**E2. Please indicate how much you agree or disagree with each of the following statements.**

|  | Strongly disagree | Disagree | Neutral | Agree | Strongly agree |
| --- | --- | --- | --- | --- | --- |
| I prefer to get *as much information as possible* about treatment options | 🞏 | 🞏 | 🞏 | 🞏 | 🞏 |
| I try to get my health care providers to *listen to my preferences* for my treatment | 🞏 | 🞏 | 🞏 | 🞏 | 🞏 |
| I am very active in my health care (as opposed to passive) | 🞏 | 🞏 | 🞏 | 🞏 | 🞏 |
| I take my *commitment to my treatment seriously* | 🞏 | 🞏 | 🞏 | 🞏 | 🞏 |
| I *rely on health professionals* or others to access the information for me, and then explain it to me | 🞏 | 🞏 | 🞏 | 🞏 | 🞏 |

We’d like everyone to please answer these questions, *even if you have never used the internet before*.

**E3. For each statement, please indicate which response best reflects your understanding *right now.***

|  | Strongly disagree | Disagree | Undecided | Agree | Strongly agree |
| --- | --- | --- | --- | --- | --- |
| I know *what* health resources are available on the internet | 🞏 | 🞏 | 🞏 | 🞏 | 🞏 |
| I know *where* to find helpful health resources on the internet | 🞏 | 🞏 | 🞏 | 🞏 | 🞏 |
| I know *how* to find helpful health resources on the internet | 🞏 | 🞏 | 🞏 | 🞏 | 🞏 |
| I know *how to use* the internet to answer my questions about health | 🞏 | 🞏 | 🞏 | 🞏 | 🞏 |
| I know how to use *the health information* I find on the internet to help me | 🞏 | 🞏 | 🞏 | 🞏 | 🞏 |
| I have the skills I need to *evaluate* the health resources I find on the internet | 🞏 | 🞏 | 🞏 | 🞏 | 🞏 |
| I can tell *high quality* health resources from *low quality* health resources on the internet | 🞏 | 🞏 | 🞏 | 🞏 | 🞏 |
| I feel *confident* in using information from the internet to make health decisions | 🞏 | 🞏 | 🞏 | 🞏 | 🞏 |

**E4.** **For each statement, please indicate which response best reflects your opinion and experience with your current GP.**

|  | Strongly disagree | Disagree | Neutral | Agree | Strongly agree |
| --- | --- | --- | --- | --- | --- |
| I *trust* my doctor | 🞏 | 🞏 | 🞏 | 🞏 | 🞏 |
| I feel *content* with my doctor’s treatment | 🞏 | 🞏 | 🞏 | 🞏 | 🞏 |
| I feel that I have an *ongoing relationship* with my doctor | 🞏 | 🞏 | 🞏 | 🞏 | 🞏 |
| I find it *easy to get help* from my doctor when I need it | 🞏 | 🞏 | 🞏 | 🞏 | 🞏 |
| I feel *comfortable asking questions* of my doctor | 🞏 | 🞏 | 🞏 | 🞏 | 🞏 |
| The doctor *does not explain* things to me | 🞏 | 🞏 | 🞏 | 🞏 | 🞏 |

- I do not have a regular GP.

**END OF SURVEY**

***Thank you very much for participating in our survey.***
