# Supplementary material for: Evaluating the Digital Health Experience for Patients in Primary Care: Mixed Methods Study
Source: J Med Internet Res. 2024 Apr 11;26:e50410. doi: 10.2196/50410 (PMC11046385; doi:10.2196/50410)
Supplement: Multimedia Appendix 3 [file jmir_v26i1e50410_app3.docx]

## **Table S1.** Phase 2 participant characteristics by frequency of eHealth use.

This is a Multimedia Appendix to a full manuscript published in the J Med Internet Res. For full copyright and citation information see http://dx.doi.org/10.2196/jmir.50410.

|  | | Frequency of eHealth use | | | | | *P* value**^a^** | | Effect size | |  |
| --- | --- | --- | --- | --- | --- | --- | --- | --- | --- | --- | --- |
|  | | Never, n (%) | Low/medium, n (%) | High, n (%) | Not reported, n (%) | Total, n |  | |  | |  |
|  | | | | | | |  | |  | | |
| All participants | | 151 (31.0) | 52 (10.7) | 236 (48.5) | 48 (9.9) | 487 | N/A^b^ | | N/A | |  |
| **Have you ever used eHealth?** | | | | | | | N/A | | N/A | | |
|  | Yes | 4 (1.2) | 52 (16.0) | 236 (72.4) | 34 (10.4) | 326 |  | |  | |  |
|  | No | 147 (100) | 0 (0) | 0 (0) | 0 (0) | 147 |  | |  | |  |
|  | Not reported | N/A | N/A | N/A | N/A | 14 |  | |  | |  |
| **Age^c^ (years), n (%)** | | | | | | | <.001 | | | 0.29 | |
|  | <25 | 7 (11.9) | 6 (10.2) | 42 (71.2) | 4 (6.8) | 59 |  | |  | |  |
|  | 25-44 | 25 (19.4) | 14 (10.9) | 83 (64.3) | 7 (5.4) | 129 |  | |  | |  |
|  | 45-64 | 46 (32.6) | 19 (13.5) | 62 (44.0) | 14 (9.9) | 141 |  | |  | |  |
|  | 65-74 | 33 (45.2) | 4 (5.5) | 25 (34.2) | 11 (15.1) | 73 |  | |  | |  |
|  | ≥75 | 35 (67.3) | 3 (5.8) | 9 (17.3) | 5 (9.6) | 52 |  | |  | |  |
|  | Not reported | 5 (15.2) | 6 (18.2) | 15 (45.5) | 7 (21.2) | 33 | N/A | | N/A | |  |
| **Gender** | | | | | | | <.001 | | | 0.18 | |
|  | Female | 88 (26.5) | 35 (10.5) | 179 (53.9) | 30 (9.0) | 332 |  | |  | |  |
|  | Male | 60 (42.9) | 16 (11.4) | 52 (37.1) | 12 (8.6) | 140 |  | |  | |  |
|  | Other/not reported | 3 (20.0) | 1 (6.7) | 5 (33.3) | 6 (40.0) | 15 | N/A | | N/A | |  |
| **Indigenous status^d^** | | | | | | | N/A^(b,c)^ | | | N/A^(b,c)^ | |
|  | Aboriginal and Torres Strait Islander | 1 (16.7) | 1 (16.7) | 3 (50.0) | 1 (16.7) | 6 |  | |  | |  |
|  | Not Aboriginal or Torres Strait Islander | 148 (31.4) | 51 (10.8) | 231 (48.9) | 42 (8.9) | 472 |  | |  | |  |
|  | Not reported | 2 (22.2) | 0 (0) | 2 (22.2) | 5 (55.6) | 9 | N/A | | N/A | |  |
| **State/territory** | | | | | | | <.001 | | | 0.20 | |
|  | ACT^e^ | 63 (24.6) | 36 (14.1) | 145 (56.6) | 12 (4.7) | 256 |  | |  | |  |
|  | NSW^f^ | 84 (38.9) | 16 (7.4) | 88 (40.7) | 28 (13) | 216 |  | |  | |  |
|  | Other/not reported | 4 (26.7) | 0 (0) | 3 (20) | 8 (53.3) | 15 | N/A | | N/A | |  |
| **Remoteness area** | | | | | | | <.001 | 0.16 | | | |
|  | Major cities of Australia | 70 (24.8) | 36 (12.8) | 162 (57.4) | 14 (5) | 282 |  | |  | |  |
|  | Inner regional Australia | 26 (41.3) | 3 (4.8) | 28 (44.4) | 6 (9.5) | 63 |  | |  | |  |
|  | Outer regional Australia | 52 (40.9) | 12 (9.4) | 43 (33.9) | 20 (15.7) | 127 |  | |  | |  |
|  | Other/not reported | 3 (20) | 1 (6.7) | 3 (20) | 8 (53.3) | 15 | N/A | | N/A | |  |
| **Personal income (per week)** | | | | | | | .10 | 0.13 | | | |
|  | ≥ AUD $1500 (US $979.22) | 24 (26.1) | 12 (13) | 50 (54.3) | 6 (6.5) | 92 |  | |  | |  |
|  | AUD $1000 to $1499  (US $652.81 to $978.56) | 18 (25.4) | 2 (2.8) | 45 (63.4) | 6 (8.5) | 71 |  | |  | |  |
|  | AUD $500 to $999  (US $326.40 to $652.16) | 35 (28.9) | 18 (14.9) | 58 (47.9) | 10 (8.3) | 121 |  | |  | |  |
|  | AUD $150 to $499  (US $97.92 to $325.75) | 37 (34.3) | 11 (10.2) | 48 (44.4) | 12 (11.1) | 108 |  | |  | |  |
|  | < AUD $150 (US $97.92) (includes no regular income) | 11 (31.4) | 6 (37.1) | 13 (37.1) | 5 (14.3) | 35 |  | |  | |  |
|  | Other/not reported | 26 (43.3) | 3 (5) | 22 (36.7) | 9 (15.0) | 60 | N/A | | N/A | |  |
| **Highest level of education** | | | | | | | <.001 | | | 0.23 | |
|  | Postgraduate degree/diploma | 20 (21.3) | 14 (14.9) | 52 (55.3) | 8 (8.5) | 94 |  | |  | |  |
|  | Bachelor’s degree | 20 (22.0) | 16 (17.6) | 48 (52.7) | 7 (7.7) | 91 |  | |  | |  |
|  | Diploma/advanced diploma | 19 (27.9) | 4 (5.9) | 40 (58.8) | 5 (7.4) | 68 |  | |  | |  |
|  | Certificate III/IV | 11 (22.9) | 5 (10.4) | 28 (58.3) | 4 (8.3) | 48 |  | |  | |  |
|  | Year 12 | 24 (27.9) | 7 (8.1) | 46 (53.5) | 9 (10.5) | 86 |  | |  | |  |
|  | ≤Year 11 | 45 (57.7) | 5 (6.4) | 20 (25.6) | 8 (10.3) | 78 |  | |  | |  |
|  | Other/not reported | 12 (54.5) | 1 (4.5) | 2 (9.1) | 7 (31.8) | 22 | N/A | | N/A | |  |
| **Employment status** | | | | | | | <.001 | | | 0.26 | |
|  | Employed (full-time or part-time) | 59 (21.1) | 26 (9.3) | 172 (61.6) | 22 (7.9) | 279 |  | |  | |  |
|  | Not employed, seeking work | 9 (37.5) | 4 (16.7) | 8 (33.3) | 3 (12.5) | 24 |  | |  | |  |
|  | Not employed, not seeking work | 15 (34.9) | 11 (25.6) | 14 (32.6) | 3 (7.0) | 43 |  | |  | |  |
|  | Retired | 68 (48.9) | 11 (7.9) | 41 (29.5) | 19 (13.7) | 139 |  | |  | |  |
|  | Not reported | 0 (0) | 0 (0) | 1 (50) | 1 (50) | 2 | N/A | | N/A | |  |
| **Occupation group** | | | | | | | .003^d^ | | | 0.19 | |
|  | Managers | 12 (20) | 6 (10) | 40 (66.7) | 2 (3.3) | 60 |  | |  | |  |
|  | Professionals | 39 (30.2) | 13 (10.1) | 63 (48.8) | 14 (10.9) | 129 |  | |  | |  |
|  | Technicians and trades workers | 19 (48.7) | 6 (15.4) | 12 (30.8) | 2 (5.1) | 39 |  | |  | |  |
|  | Community and personal service workers | 19 (41.3) | 7 (15.2) | 19 (41.3) | 1 (2.2) | 46 |  | |  | |  |
|  | Clerical and administrative workers | 25 (28.1) | 5 (5.6) | 52 (58.4) | 7 (7.9) | 89 |  | |  | |  |
|  | Sales workers | 10 (37.0) | 2 (7.4) | 7 (25.9) | 8 (29.6) | 27 |  | |  | |  |
|  | Machinery operators and drivers | 6 (60) | 1 (10) | 1 (10) | 2 (20) | 10 |  | |  | |  |
|  | Laborers | 4 (44.4) | 1 (11.1) | 3 (33.3) | 1 (11.1) | 9 |  | |  | |  |
|  | Other/not reported | 17 (21.8) | 11 (14.1) | 39 (50.0) | 11 (14.1) | 78 | N/A | | N/A | |  |
| **Marital status** | | | | | | | .02^g^ | | | 0.15 | |
|  | Never married | 24 (19.8) | 16 (13.2) | 73 (60.3) | 8 (6.6) | 121 |  | |  | |  |
|  | De facto | 14 (25) | 7 (12.5) | 32 (57.1) | 3 (5.4) | 56 |  | |  | |  |
|  | Married | 71 (34) | 21 (10) | 94 (45) | 23 (11) | 209 |  | |  | |  |
|  | Separated but not divorced | 5 (41.7) | 1 (8.3) | 5 (41.7) | 1 (8.3) | 12 |  | |  | |  |
|  | Divorced | 18 (40.9) | 3 (6.8) | 19 (43.2) | 4 (9.1) | 44 |  | |  | |  |
|  | Widowed | 16 (48.5) | 4 (12.1) | 11 (33.3) | 2 (6.1) | 33 |  | |  | |  |
|  | Other/not reported | 3 (25) | 0 (0) | 2 (16.7) | 7 (58.3) | 12 | N/A | | N/A | |  |
| **Country of birth** | | | | | | | .18 | | | 0.09 | |
|  | Australia | 110 (30.6) | 31 (8.6) | 180 (50.1) | 38 (10.6) | 359 |  | |  | |  |
|  | Other Organisation for Economic Cooperation and Development countries | 24 (41.4) | 7 (12.1) | 25 (43.1) | 2 (3.4) | 58 |  | |  | |  |
|  | Non–Organisation for Economic Cooperation and Development countries | 14 (35) | 8 (20) | 18 (45) | 0 (0) | 40 |  | |  | |  |
|  | Not reported | 3 (10) | 6 (20) | 13 (43.3) | 8 (26.7) | 30 | N/A | | N/A | |  |
| **Language spoken at home** | | | | | | | .01 | | | 0.14 | |
|  | English only | 131 (30.8) | 39 (9.2) | 215 (50.6) | 40 (9.4) | 425 |  | |  | |  |
|  | Language other than English | 17 (34.7) | 11 (22.4) | 18 (36.7) | 3 (6.1) | 49 |  | |  | |  |
|  | Not reported | 3 (23.1) | 2 (15.4) | 3 (23.1) | 5 (38.5) | 13 | N/A | | N/A | |  |

^a^Comparing frequency of eHealth use across subgroups. Excludes not reported categories.

^b^N/A: not applicable.

^c^Cell sizes insufficient for chi-square test.

^d^Sales workers, machinery operators and drivers, and laborers are combined for significance testing.

^e^ACT: Australian Capital Territory.

^f^NSW: New South Wales.

^g^Separated but not divorced, and divorced combined for significance testing.
